# Hemodynamic Changes Associated with Two Different Concentrations of Adrenaline in Lignocaine Solution: A Comparative Analysis

**DOI:** 10.7759/cureus.4434

**Published:** 2019-04-11

**Authors:** Abu Mohammed Shakeel, Pradeep J Christopher, Srivatsa Kengasubbiah, Senthil Kumar, Vandana Shenoy

**Affiliations:** 1 Oral and Maxillofacial Surgery, Thai Moogambigai Dental College & Hospital, Chennai, IND

**Keywords:** lignocaine, adrenaline, oxygen saturation, pulse rate, blood pressure

## Abstract

Local anesthetic (LA) agents are commonly employed during surgical procedures in dentistry to control pain. There are a variety of LA agents available in the market with different concentration of adrenaline added to increase the efficacy. The systemic effects of adrenaline used as vasoconstrictors in LA solution are short lived or transient in nature; however, there are instances of associated cardiovascular problems. This study compares the efficacy and cardiovascular effects with the use of 2% lignocaine with two different concentrations of adrenaline.

## Introduction

Local anesthetic (LA) are chemical solutions that reversibly block the transmission of the action potential of nerve membranes [1]. There are a variety of LAs available in the market, lignocaine 2% is considered gold standard [2]. Lignocaine diffuses readily through interstitial tissues and lipid-rich nerves, giving rapid onset of action. Its vasodilating effect is more than that of prilocaine and mepivacaine [3]. Lignocaine is a potent vasodilator and the anesthetic effect is short-lived when employed alone.

Adrenaline as a vasoconstrictor additive shortens the onset time, prolongs the duration as well as the depth of anesthesia. It is effective in preventing or minimizing blood loss during surgical procedures. Due to its peripheral vaso-constrictive effect, adrenaline delays the absorption of LA and its systemic toxicity is reduced. Adrenaline can be safely employed at 0.2 mg dose in healthy patients and it is best to limit the total dose to 0.04 mg in cardiac patients. Adrenaline acts directly on both α and β-adrenergic receptors. Systemically adrenaline-like drugs can cause a number of cardiovascular disturbances while most are transient, permanent injury or untoward cardio-vascular and cerebro-vascular accidents may occur [4-5].

## Materials and methods

The objectives of the study were the following:

1. To study the cardiovascular effects of LA with adrenaline in two different concentrations - 1:80000 and 1:200000

2. To compare the efficacy of 2% lignocaine with two different concentrations of adrenaline - 1:80000 and 1:200000

3. To study the impact and changes associated with oxygen saturation in different adrenaline concentration 1:80000 and 1:200000

Eighty cases that required extraction of maxillary and/or mandibular teeth were selected and allocated to Group 1 and Group 2 randomly in patients requiring bilateral extractions. Unilateral extractions were carried out in a single sitting and the other side was completed on the next visit. All the cases were carried out by a single operator.

The following data were collected during the study:

1. Time of administration of LA

2. The onset of anesthesia noted as subjective and objective symptoms (subjective: tingling and numbness in the lower lip and tongue; objective: absence of pain on instrumentation)

3. Amount of LA used The pulse rate, blood pressure (BP), and oxygen saturation were recorded using an automated multi-nodular monitor. This was done before and immediately after the administration of LA, after extraction, and 15 mins post-operatively. The evaluation of analgesia was done by the operator and recorded as 'successful', 'partial success', and 'failure'. Any sign of systemic toxicity was recorded.

## Results

The safety and efficacy of the two solutions with two different concentrations of adrenaline were studied in 80 patients randomly by dividing them into two groups. The first patient was allocated by draw to Group 1 and all the other patients were divided randomly between the two groups. Group 1 patients received 2% lignocaine with 1:80000 adrenaline, while Group 2 patients received 2% lignocaine with 1:200000 adrenaline. The age varied from 18 to 71, while the mean age was 35.

There was no significant change in both the groups from the point of view of the time of onset. With regard to the duration of action of LA, the anesthetic duration of 1:80000 adrenaline concentrations was longer than that of 1:200000. This is due to the faster absorption of LA when used with less concentration of adrenaline. The amount of LA used for both the groups was almost the same.

**Figure 1:**
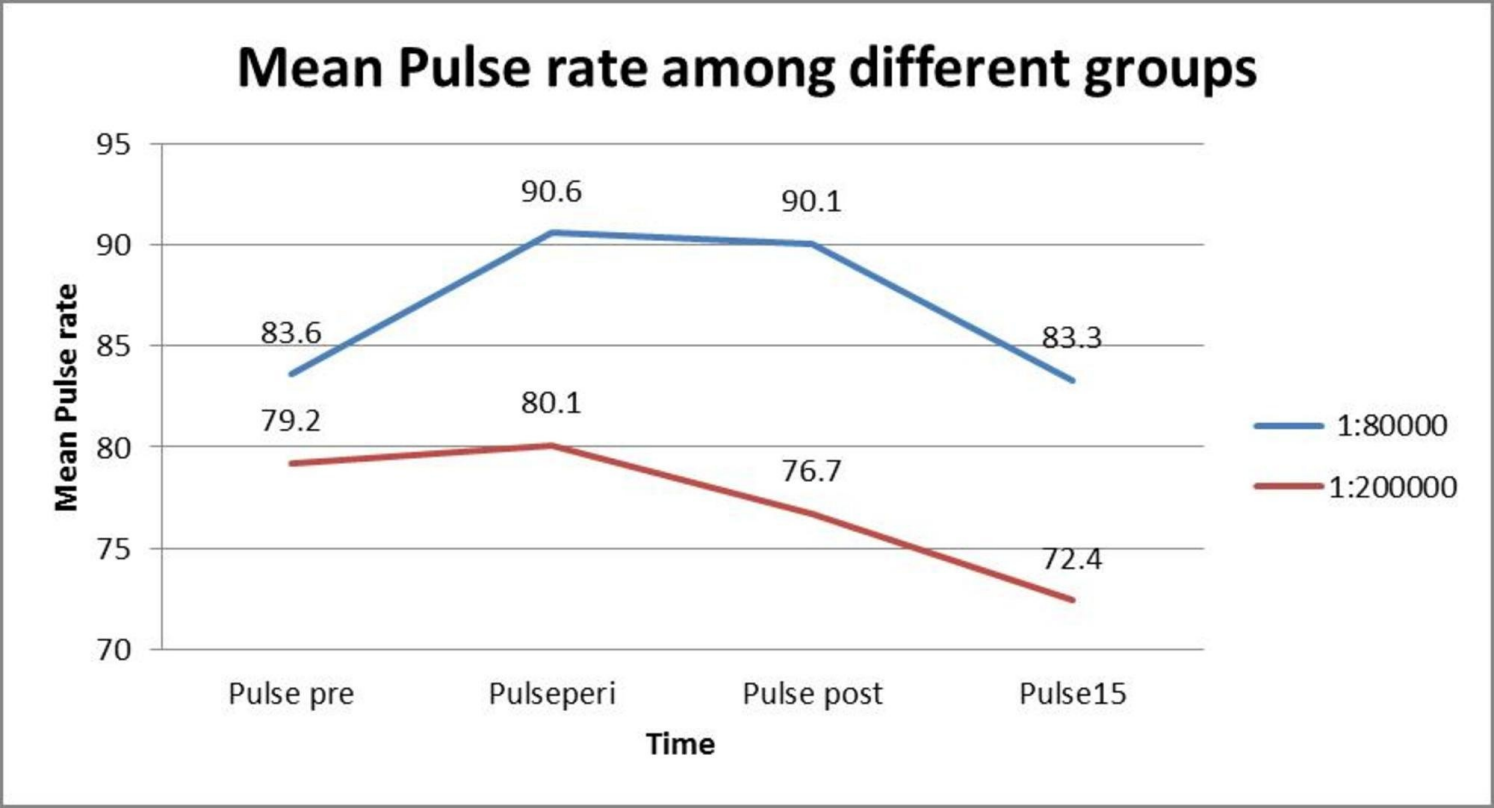
Mean pulse rate in both groups Pulse pre = Before the administration of LA; Pulseperi = Immediately after the administration of LA; Pulse post = After the extraction; Pulse 15 = 15 mins post-operatively.

There was a significant rise in the pulse rate immediately after LA with 1:80000 adrenaline concentrations was used; it dropped to the normal rate gradually after 15 min as seen in Figure 1. But when LA with 1:200000 adrenaline concentrations was used, there was no significant rise in the pulse rate.

**Figure 2:**
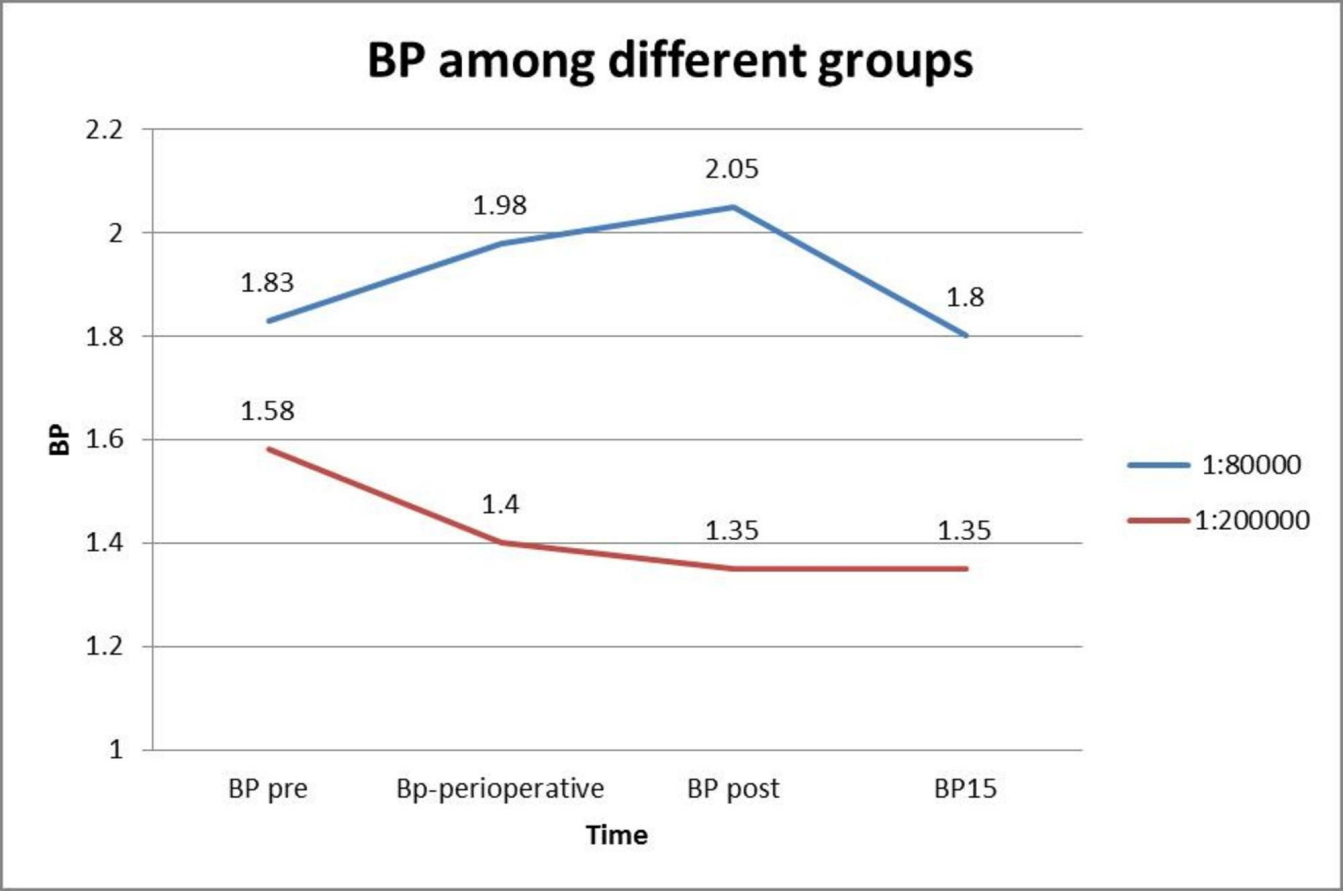
Blood pressure in both groups BP pre = Before the administration of LA; BP perioperative = Immediately after the administration of LA; BP post = After the extraction; BP15 = 15 mins post-operatively.

While assessing the BP, there was significant rise when LA with 1:80000 adrenaline concentrations was used, whereas there was no major change observed when LA with 1:200000 was used (Figure 2).

**Figure 3:**
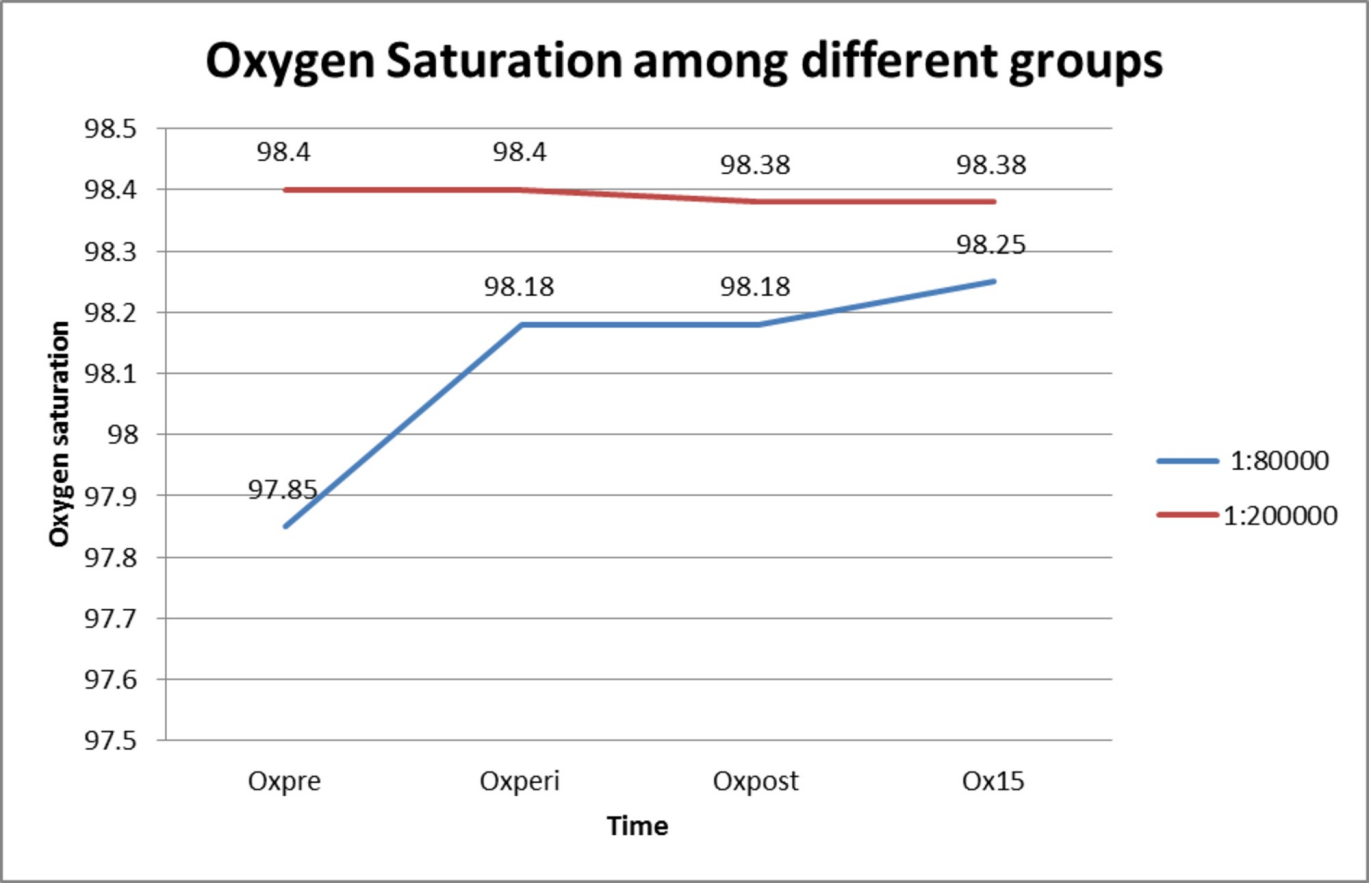
Oxygen saturation in both groups Oxpre = Before the administration of LA; Oxperi = Immediately after the administration of LA; Oxpost = After the extraction; Ox15 = 15 mins post-operatively.

There was no significant change in oxygen saturation in both groups as shown in Figure 3.

## Discussion

Lignocaine is the most widely used anesthetic in dentistry. Lignocaine is considered the gold standard to which other anesthetics are often compared. Lignocaine can be used alone or in combination with a vasoconstrictor; used alone, it does not have a long duration of anesthetic effect majorly due to its potent vasodilation property. To overcome this drawback, vasoconstrictors are added in the LA solution [6]. Vasoconstrictors employed in LA solutions have the potential for interacting with a wide variety of drugs [7].

Physiological responses associated with LA solutions containing a adrenaline have included changes in heart rate and BP, dysarrythmias, ischemic changes (ST segment and T wave), the release of endogenous catecholamines, endocrine response to surgery, and hypokalemia [8] LA agents with adrenaline as the vasoconstrictor used for the surgical soft tissue and bone interventions in the oral region tend to cause more post-operative pain than LA without adrenaline as the vasoconstrictor [9].

Significant cardiovascular effects were observed in the study as seen in the statistical analysis; there was a significant rise in the mean pulse rate when 1:80000 adrenaline was used; whereas, no significant change was observed when 1:200000 was used. There was a significant rise in the systolic and diastolic BP when LA with 1:80000 adrenaline was used while 1:200000 adrenaline group did not show any significant change [10-11]. The time of onset of anesthesia as well as the amount of LA used in our study is similar to the study conducted by Malamed et al. [12]. Gregorio et al. have reported that it is important to stress that with articaine and other local anesthetic solutions in general, 1:100000 and 1:50000 epinephrine concentrations are associated with greater cardiovascular stimulation than 1:200000 epinephrine formulations [13].

For an adult healthy patient, LA with any of the concentration of adrenaline can be used as the efficacy is not altered in both the solutions. But in elderly and cardiac patients, LA with 1:200000 adrenaline concentration may be preferred. Since the duration of anesthesia is significantly different for both the solutions, LA with 1:80000 is preferred in the case of long procedures.

## Conclusions

The present study of two types of 2% lignocaine with two different concentrations showed that both of them have the same efficacy. Coming to the cardiovascular effects, 1:80000 adrenaline concentrations showed a significant rise in pulse rate as well as BP when compared with the other drug. For cardiac and elderly patients, 1:200000 adrenaline concentration may be recommended as it showed better cardiac stability.

## References

[1] Caviedes-Bucheli J, Rojas P, Escalona M (2009). The effect of different vasoconstrictors and local anesthetic solutions on substance P expression in human dental pulp. J Endod.

[2] Kanaa MD, Whitworth JM, Corbett IP, Meechan JG (2006). Articaine and lidocaine mandibular buccal infiltration anesthesia: a prospective randomized double-blind crossover study. J Endod.

[3] Malamad SF (1997). Handbook of Local Anesthesia.

[4] Yagiela JA (1999). Adverse drug interactions in dental practice: interactions associated with vasoconstrictors. J Am Dent Assoc.

[5] Haase A, Reader A, Nusstein J, Beck M, Drum M (2008). Comparing anesthetic efficacy of articaine versus lidocaine as a supplemental buccal infiltration of the mandibular first molar after an inferior alveolar nerve block. J Am Dent Assoc.

[6] Hersh EV, Hermann DG, Lamp CJ, Johnson PD, Macafee KA (1995). Assessing the duration of mandibular soft tissue anesthesia. J Am Dent Assoc.

[7] Yagiela JA, Duffin SR, Hunt LM (1985). Drug interactions and vasoconstrictors used in local anesthetic solutions. Oral Surg Oral Med Oral Pathol.

[8] Elad S, Admon D, Kedmi M (2008). The cardiovascular effect of local anesthesia with articaine plus 1:200,000 adrenalin versus lidocaine plus 1: 100,000 adrenalin in medically compromised cardiac patients: a prospective, randomized, double blinded study. Oral Surg Oral Med Oral Pathol Oral Radiol Endod.

[9] Hanvold KI, Vigen EC, Jorkjend L, Aass AM, Skoglunda LA (2008). Increase in volume of dental local anaesthetic solution while maintaining the tissue lidocaine and adrenaline concentration does not increase acute postoperative pain after gingivectomy. Br J Oral Maxillofac Surg.

[10] Dagher FB, Yared GM, Machtou P (1997). An evaluation of 2% lidocaine with different concentrations of epinephrine for inferior alveolar nerve block. J Endod.

[11] Malamed SF, Gagnon S, D Leblanc (2000). Efficacy of articaine: a new amide local anesthetic. J Am Dent Assoc.

[12] Santos CF, Modena KC, Giglio FPM (2007). Epinephrine concentration (1:100,000 or 1:200,000) does not affect the clinical efficacy of 4% articaine for lower third molar removal: a double-blind, randomized, crossover study. J Oral Maxillofac Surg.

[13] Gregorio LV, Giglio FP, Sakai VT (2008). A comparison of the clinical anesthetic efficacy of 4% articaine and 0.5% bupivacaine (both with 1:200,000 epinephrine) for lower third molar removal. Oral Surg Oral Med Oral Pathol Oral Radiol Endod.

